# Dipeptidyl peptidase-4 inhibitor: Sitagliptin down-regulated toll-like receptor 4 signaling pathway to reduce uterine injury in rats

**DOI:** 10.22038/IJBMS.2022.64552.14202

**Published:** 2022-11

**Authors:** Remon Roshdy Rofaeil, Sabreen Mahmoud Ahmed, Haitham Ahmed Bahaa, Ahmad Mahran, Nermeen N. Welson, Walaa Yehia Abdelzaher

**Affiliations:** 1 Department of Pharmacology, Faculty of Medicine, Minia University, Minia, Egypt; 2 Department of Pharmacology, Faculty of Pharmacy, Deraya University, Minia, Egypt; 3 Depatment of Human Anatomy and Embryology, Faculty of Medicine, Minia University, delegated to Deraya University-New Minia City, Egypt; 4 Department of Obstetrics and Gynecology, Faculty of Medicine, Minia University. Minia, Egypt; 5 Department of Forensic Medicine and Clinical Toxicology, Faculty of Medicine, Beni-Suef University, Beni-Suef, Egypt

**Keywords:** Anti-apoptotic, Anti-inflammatory, Sitagliptin, Toll-like receptor 4, Uterine ischemia

## Abstract

**Objective(s):**

Uterine ischemia is a common problem with ongoing controversy about its pathogenesis and prevention. The present study aimed to investigate the protective role of sitagliptin against uterine ischemia-reperfusion injury (IRI).

**Materials and Methods:**

Rats were allocated into 4 groups: control, sitagliptin (SIT) (5 mg/kg), IR; ischemia was induced followed by reperfusion, and IR+SIT; SIT was administered 1 hr before IRI. Uteri were removed for histopathological and biochemical observations. Malondialdehyde (MDA), total nitrites (NOx), reduced glutathione (GSH), superoxide dismutase (SOD) activity, tumor necrosis factor-α (TNF-α), interleukin-6 (IL-6), and toll-like receptor 4 (TLR4) were all measured. Hematoxylin and eosin (H&E) stain, Periodic acid-Schiff stain (PAS), and caspase-3 immunostaining were applied.

**Results:**

In the IR+SIT group; NOx, GSH, and SOD activities increased significantly. Meanwhile, the levels of MDA, TNF-α, IL-6, TLR4, and caspase-3 immunoexpression showed a significant reduction, as compared with the IR group. In the IR+SIT group, an improvement in the histopathological picture was noticed.

**Conclusion:**

The results showed that sitagliptin confers protection against uterine IRI through anti-oxidant, anti-inflammatory, and anti-apoptotic effects with a possible role for TLR4.

## Introduction

One of the common problems in gynecology and obstetrics is the temporary ischemia of the uterus. There is a heated debate about the pathophysiology of uterine ischemia and how to prevent its occurrence and complications (1, 2).

The process of ischemia-reperfusion injury is multifactorial and multiple mechanisms are involved in its pathogenesis. Ample evidence indicates that damage is caused in part by the excessive production of reactive oxygen species or free radicals. As a result, free radicals provoke inflammatory responses, possibly affecting distant organs. It can cause remote organ damage (3).

The detrimental effect of IRI occurs as ischemia results in oxygen supply reduction with cell energy depletion together with toxic metabolite accumulation, favoring the occurrence of oxidative stress and apoptosis. Thereafter, with reperfusion, enhanced generation of nitrogen species and reactive oxygen species (ROS) occurs with simultaneous interaction with lipids, nucleic acids, and proteins, which enhances membrane lipid peroxidation. Grievously, these events result in tissue injury, loss of cell function, cell structure disorganization, inflammation, and apoptosis (4-6).

Toll-like receptors (TLRs) are a family of transmembrane proteins that are widely expressed in various tissues. Many studies have reported their involvement in creating an inflammatory response in IRI, mostly through their interaction with endogenous agonists. One of these TLRs is TLR4, which is widely expressed in the uterus (7, 8).

Sitagliptin (SIT), an inhibitor of dipeptidyl peptidase-4 (DPP-4) mainly used for type II diabetes mellitus, was reported to antagonize IR injury in the liver (9), kidney (10), intestine (11), and testis (4). Such a protective effect is mediated through antioxidant, anti-apoptotic, and anti-inflammatory mechanisms. Furthermore, a promising therapeutic effect of SIT on an *in vitro* model was reported with amelioration of hypoxia-induced oxidative stress in endometrial stromal cells (12).

The objective of the current study was to evaluate the influence of SIT on uterine IR injury in rats’ uteri.

## Materials and Methods

***Ethics***

All experimental protocols were approved by the Institutional Ethical Committee, Faculty of Medicine, Minia University, Egypt (Approval No. 29:3/2021). This study is reported in accordance with ARRIVE guidelines. All experiments were performed following relevant guidelines and regulations.

***Chemicals***

SIT was purchased from Multipharma, Egypt. Tumor necrosis factor-alpha (TNF-α) was quantified using an ELISA kit (Elabscience, USA). TLR4 and interleukin-6 (IL-6) were measured by an ELISA kit (Cusabio, USA). Reduced glutathione (GSH) kit was obtained from Biodiagnostic, Egypt.

***Animals and experimental design***

**Figure 1:**
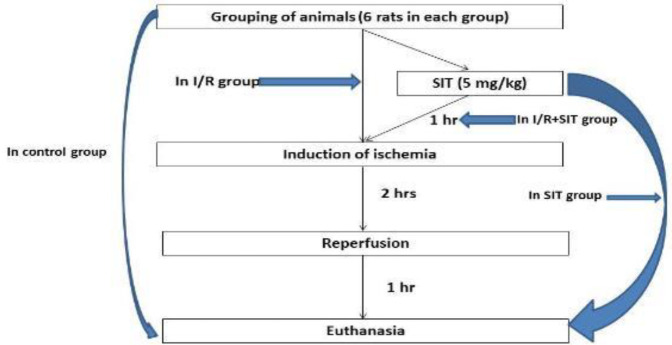
Schematic representation of the timing of the work steps

The study was performed on 24 female Wistar albino rats (8 to 10 weeks old) weighing 220-270 g. They were obtained from the National Research Center, Cairo, Egypt. The animals were fed *ad libitum*, housed individually in steel cages in a temperature-controlled environment (23±2 ^°^C), and exposed to a 12 hr/12 hr light/dark cycle. All animals were in the diestrus phase of the estrous cycle, as confirmed by vaginal smears (13). All rodents were randomly assigned to one of four groups (6 rats each): control group, which received saline (IP), SIT group, which received SIT (5 mg/kg, IP) (14), IR group, which underwent IRI only, and IR+SIT group, which received SIT (5 mg/kg, IP) 1 hr before IR. SIT was prepared as a powder dissolved in saline at 25 ^°^C (Figure 1).

Rats were weighed, ketamine (50 mg/kg) and xylazine (10 mg/kg) were given, and when necessary, the drugs were repeated to maintain anesthesia. Preoperative sterilization was done, and a midline laparotomy incision was performed in the lower abdomen. We exposed the abdominal aorta. Thereafter, a clamp was placed above the iliac artery bifurcation and above and below the ovaries (1 cm) to close collateral circulation. Then we closed the abdomen, and a moist dressing covered the wound, aiming to reduce heat loss (15). In the control and SIT groups, laparotomy was done without IR induction. In the IR group, ischemia was induced for 2 hr, followed by reperfusion for a 1 hr duration. In the IR+SIT group, SIT was given 1 hr before IR. 

***Tissue sampling***

At the end of the experiment, uterine horns were dissected, the right one was assigned for histopathologic examination, and the left one was frozen at -80 ^°^C for the biochemical tests.

***Biochemical determination***

*Determination of oxidative stress parameters*

Homogenization was done in potassium phosphate buffer, 10 mM pH (7.4). Homogenates were centrifuged (4000 g at 4 ^°^C for 10 min). In addition, malondialdehyde (MDA), total nitrites (NOx), reduced glutathione (GSH), and superoxide dismutase (SOD) were measured in the supernatants. As a marker of lipid peroxidation, MDA levels were quantified as described by Buege and Aust (16). SOD was determined by Marklund and Marklund (17). Total nitrites were estimated through the reduction of nitrate into nitrite followed by color development with Griess reagent in an acidic medium (18). GSH was estimated using colorimetric kits following their instructions

*Determination of inflammatory mediators and TLR4*

Uterine TNF-α, IL-6, and TLR4 were measured using ELISA kits following the manufacturer’s instructions.

***Histologic evaluation***

*Histological and immunohistochemical studies*

The uterus was fixed in 10% formal-saline and then processed to paraffin blocks, from which 5-mm-thick sections were cut and stained with Hematoxylin and eosin (H&E), Periodic acid-Schiff (PAS) (19), and caspase-3 immunostaining (20).

*Morphometric study*

Measurements were performed in five non-overlapping fields from five different sections of five different rats in each group at ×100 magnifications, using an image analyzer (Leica Imaging System, Germany) to measure: 

(a) Area% of the PAS-positive material in PAS-stained sections.

(b) Area% of the immunopositive reaction of caspase-3.

(c) Mean thickness of the endometrial surface epithelium in um.

***Statistical analysis***

All values are presented as means±standard deviation (SD). One-way analysis of variance (ANOVA) followed by the Tukey–Kramar post-analysis test was used to analyze the results. *P*-values less than 0.05 were considered significant. Graph Pad Prism was used for statistical calculations (version 5.01 for Windows, Graph Pad Software, San Diego, California, USA, and www.graphpad.com)**.** 

## Results

***Effect of sitagliptin on oxidative stress parameters***

**Figure 2:**
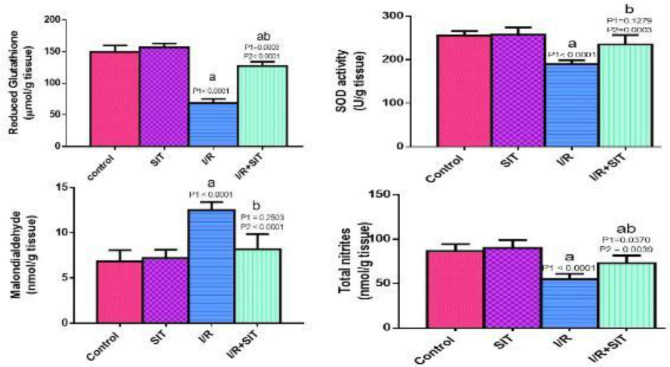
Effect of sitagliptin on oxidative stress parameters

In the IR group, MDA significantly increased as compared with the control group. At the same time, NOx, GSH, and SOD were decreased in the IR group as compared with the control group. In contrast, in the IR+SIT group, MDA was reduced. Meanwhile, NOx, GSH, and SOD were increased as compared with the IR group, as shown in Figure 2. 

***Effect of sitagliptin on inflammatory parameters***

**Figure 3:**
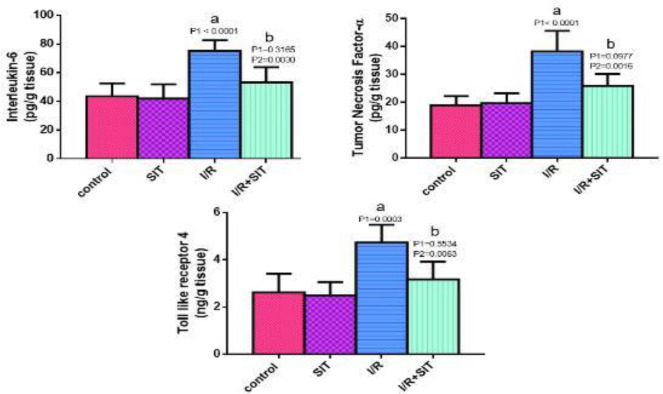
Effect of sitagliptin on interleukin-6, tumor necrosis factor-α, and Toll-like receptor 4

TNF-α and IL-6 levels were higher in the IR group than in the control group. As shown in Figure 3, TNF-α and IL-6 levels were significantly lower in the IR+SIT group compared with the IR group. 

***Effect of sitagliptin on toll-like receptor 4***

In the IR group, TLR4 was increased as compared with the control. As shown in Figure 3, there was a significant reduction of TLR4 in the IR+SIT group compared with the IR group. 

***Histological results***

*H&E-stained sections*

**Figure 4:**
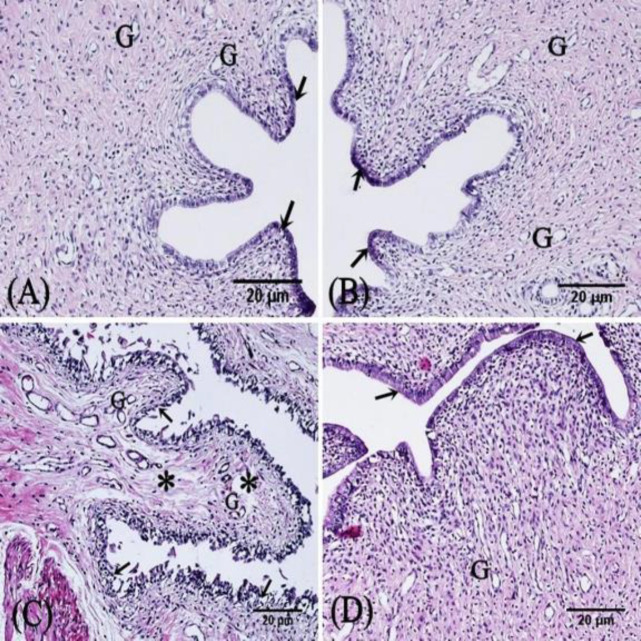
Photomicrographs of sections in the rat endometrium

**Figure 5:**
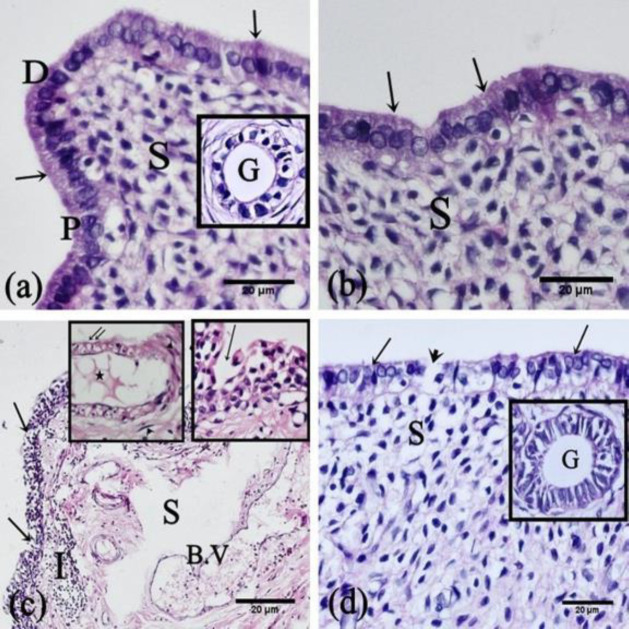
Photomicrographs of sections of the rat endometrium

Histological examination of the uterine sections of the control rats, and SIT-treated rats revealed that the endometrium consisted of columnar epithelium with dark and pale secretory cells covering a thick stroma. The stroma enclosed stromal cells, uterine glands, and blood vessels (Figure 4a, 4b, and Figure 5a, 5b). In the uterine sections of IR rats, the endometrial histology was disturbed; the epithelium was sloughed, the stroma showed disorganized cellular arrangement with cellular infiltration, and dilated congested blood vessels. Most of the glands were atrophied with retained secretions in the lumens (Figure 4c and Figure 5c). The uterine sections of IR+SIT-treated rats displayed almost normal endometrial structure except in limited regions with focal epithelial disruptions as shown Figure 4d and Figure 5d.

*PAS-stained sections*

**Figure 6:**
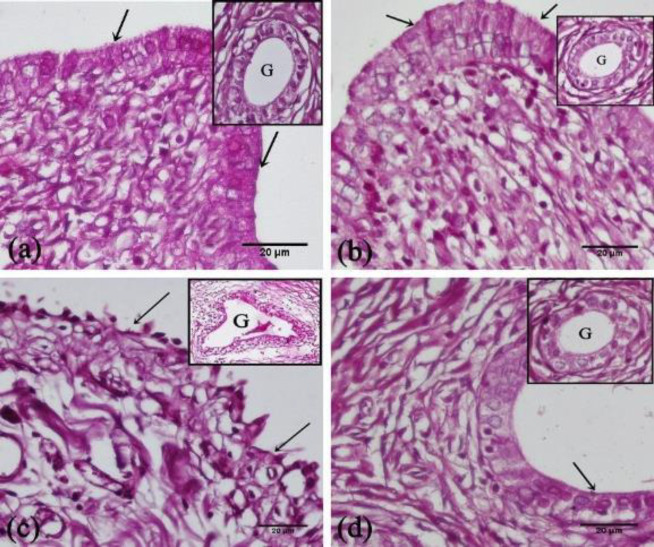
Photomicrographs of the PAS reaction in the rat endometrial sections

The uterine sections of the control rats and SIT-treated rats displayed PAS-positive regular coats on the surface epithelium and around the stromal glands (Figures 6a and 6b). While the uterine sections of the IR rats revealed loss of PAS reaction in the disrupted epithelium and atrophied glands (Figure 6c). However, the sections of IR+SIT-treated rats displayed moderate PAS reactions in the columnar epithelium and glands as shown in Figure 6d.

***Immunohistochemically stained sections***

**Figure 7:**
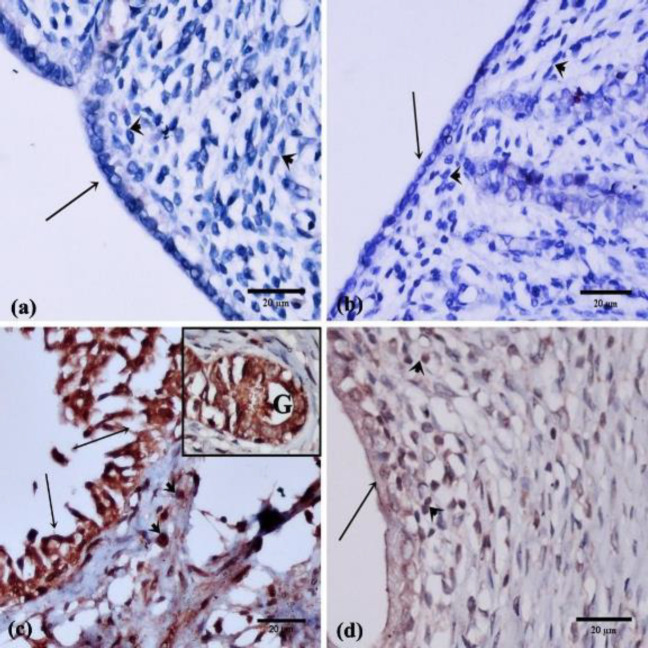
Photomicrographs of caspase-3 cytoplasmic immunoreaction in the rat endometrial sections

In the control and SIT-treated groups, endometrial sections displayed negative immunoreactions for caspase-3 in the cytoplasm of endometrial epithelium and stroma cells (Figures 7a and 7b). In contrast, endometrial sections of the IR rats displayed positive cytoplasmic immunoreactions for caspase-3 in the degenerated epithelium, stroma, and atrophied glands (Figure 7c). In the IR+SIT-treated group, endometrial sections of rats displayed weak immunoreactions for caspase-3 in the cytoplasm of some epithelial and stromal cells as shown in Figure 7d. 

***Morphometric results***

**Figure 8:**
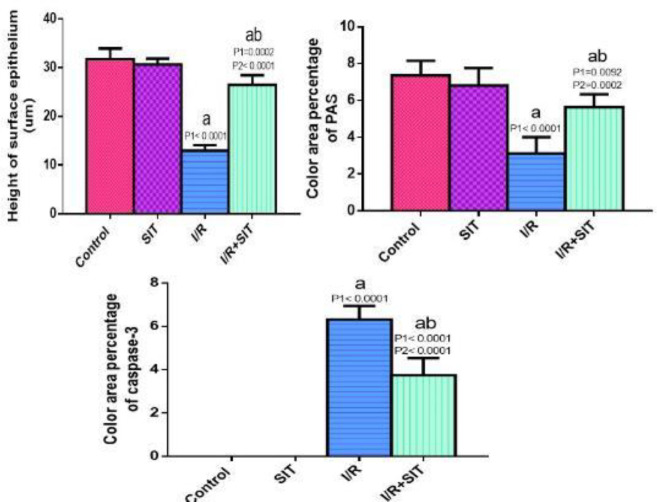
The histopathologic scores of the uterus

The mean area % for PAS, the mean area % for caspase-3 immunostaining, and the mean thickness of the endometrial epithelium showed a statistically significant difference among the groups. The scores of caspase-3 immunostaining were significantly higher for the IR group as compared with the other groups. Whereas the scores of the mean color area percentage of PAS and the mean thickness of the endometrial epithelium were significantly lower for the IR-treated group when contrasted with the other groups as shown in Figure 8.

SIT: sitagliptin; I/R: ischemia/reperfusion

Results represent mean±SD (n=6). ^a^ Significant difference from the control group, ^b ^Significant difference from the IR group. Significance is at *P<*0.05. P1: significance from the control group and P2: significance from the I/R group

SOD: superoxide dismutase

Results represent mean±SD (n=6). ^a^ Significant difference from the control group, ^b^ Significant difference from the IR group. Significance is set at *P<*0.05. P1: significance from the control group and P2: significance from the I/R group

(a) Group (I) & (b) Group (II) show intact columnar epithelium (arrow) and the underlying stroma with endometrial gland (G)

(c) Group (III) shows epithelial degeneration (arrow), stroma with empty spaces (*), and atrophied endometrial gland (G)

(d) Group (IV) shows an intact columnar epithelium (arrow) and underlying stroma with endometrial gland (G)

(H&E, X100)

(a) Control group shows columnar epithelial cells (arrow) with dark (D) and pale (P) nuclei and underlying stroma (S). Inset: endometrial gland (G)

(b) The SIT group shows intact columnar epithelium (arrow) and underlying stroma (S)

(c) The IR group shows epithelial degeneration (arrow), stroma with empty spaces (S), cellular infiltration (I), and congested dilated blood vessels (B.V). Insets: epithelial loss (arrow), atrophied endometrial gland (double arrows) with retained secretion (star), and fibroblasts (arrowhead) around

(d) The IR+SIT group shows intact columnar epithelium (arrow) with focal disruption (arrowhead) and underlying stroma (S). Inset: endometrial gland (G)

(H&E; a, b, d X 400, c X200, and insets X400)

Endometrial epithelium (arrow) and around glands (G)

(a & b) From the control and SIT groups, respectively, show a strong positive reaction. (c) From the IR group, showing loss of reaction. (d) From the IR+SIT group showing a moderate reaction

(PAS, X400)

Endometrial epithelium (arrow), stroma cells (arrowhead), and glands (G)

(a & b) From the control and SIT groups, respectively, show negative immunoreactivity. (c) From the IR group, showing positive immunoreaction. (d) From the IR+SIT group showing weak immunoreaction

(Caspase-3 immunostaining, X400)

Results represent the mean±SD (n=6). ^a^ Significant difference from the control group, ^b ^Significant difference from the IR group. Significance is set at *P<*0.05. P1: significance from the control group and P2: significance from the I/R group

PAS: periodic acid chief stain

## Discussion

Uterine ischemia is a common problem with ongoing controversy about its pathogenesis, prevention, and management (1, 2, 15). In this study, the ameliorative effect of SIT against uterine IRI was investigated.

As a result of IR, tissue damage occurs due to the massive production of ROS, which is synthesized continuously inside the cells as a result of biochemical reactions and external factors. ROS are highly reactive free radicals, including superoxide, hydrogen peroxide, hydroxyl radical, and peroxynitrite, that damage DNA, RNA, and cellular proteins, resulting in cellular dysfunction and death. In IRI, an imbalance occurs between ROS production and the antioxidant defense of the cell, creating an oxidative stress status in cells (21). Furthermore, the ischemic injury disrupts the cell membrane integrity, causing the release of cellular contents as inflammatory mediators. Interleukins are associated with ischemia-induced inflammation as they activate caspases, resulting in apoptosis and cell death (22).

In the current study, the IR group showed induction of oxidative stress evidenced by the elevation in MDA and reduction in NO, GSH, and SOD levels. It also caused inflammation, as evidenced by the increase in TNF-α and IL-6. All these changes are in agreement with several previous studies (15, 21-23).

In contrast, SIT administration before IR ameliorated all these changes, which is in agreement with other studies that stated the anti-oxidant and anti-inflammatory actions of SIT in different IR models; hepatic, renal, cerebral, intestinal, and cardiac (9, 10, 24-26).

In the present study, the sections obtained from the control and SIT-treated groups showed the surface columnar epithelium and the underlying stroma with no pathological changes. Rats of the IR group revealed sloughed epithelium, disorganized stroma, cellular infiltration, and atrophied glands. The current results were in coincidence with those of some authors who proved the same findings (27, 28).

Running in the same stream, a significant elevation in the caspase-3 immunoreaction was detected in IR rats as compared with the control group. This can be attributed to the fact that caspase-3 activation by I/R injury elevates Bax, prevents Bcl-2 expression, and forms Bax/Bcl-2 heterodimers which induce apoptosis (29).

Morphometrically, a significant decrease in the mean endometrial thickness and the mean percentage area of PAS was observed in the IR group compared with the control group. So, IR adversely affects the uterine morphology, and this is consistent with Hu and Yuan, who reported that IR induces hypoxia, which in turn leads to apoptosis that is involved in the pathogenesis of reduced endometrial thickness (30).

On the other hand, SIT administered before IR improved the histopathological picture, reduced caspase-3 immunoreaction, and increased both mean endometrial thickness and mean percentage area of PAS as compared with the IR group. These actions were proposed to be due to their ability to modify the antioxidant activity, reduce the biomarkers of oxidative stress, antagonize inflammation and reduce apoptosis (9).

TLR-4 was shown to have a critical role in the pathogenesis of renal IRI. Although it was identified for its specific binding to LPS, one of the gram-negative bacterial cell wall components, it has recently been proven to be activated by mediators that are released by stressed and necrotic cells, as well as degraded products of endogenous macromolecules. Furthermore, it acts as a monitoring receptor used for tissue injury detection (31-33).

Wu and his colleagues reported that TLR4 induces the transcription of inflammatory genes such as IL-6, IL-1β, and TNF-α. Moreover, TLR4 causes the overexpression of macrophage inflammatory protein-2 (MIP-2) and monocyte chemoattractant protein-1 (MCP-1) by resulting in neutrophil and macrophage recruitment. Cytokine release starts with ischemia and is aggravated upon reperfusion. Surprisingly, in IRI models of TLR-4-deficient animals, no such overexpression was noticed, no increase in TNF-α, and there was a limited elevation in IL-6, IL-1β, and MCP-1 protein levels (34). It also facilitates leukocyte migration and infiltration, which were absent in IRI models of TLR-4 knockout animals (35). TLR4 also induces apoptosis in IRI models (36, 37).

In the IR group, inflammation, oxidative stress, and apoptosis were accompanied by an increase in TLR4, which is in accordance with numerous reports. They stated that an increase in TLR4 occurred in IRI in different organs through induced oxidative stress, inflammation, and apoptosis (38, 39, 40). In contrast, in the IR+SIT group, there was a reduction in TLR4. This reduction might be linked to the antioxidant, anti-inflammatory, and anti-apoptotic actions of SIT in uterine IR injury.

## Conclusion

SIT was found to confer protection against uterine IRI through antagonizing oxidative stress, inflammation, and apoptosis, with a possible role for TLR4.

## Authors’ Contributions

RRR and WYA Designed and performed the experiment, data analysis, and supervision. SMA Performed histological and immunohistochemical examinations. NNW Cooperated in performing the experiment and wrote the final draft. HAB and AM Cooperated in writing the primary draft. All authors helped revise the manuscript. 

## Funding sources

This study did not receive any specific funding from funding agencies in the public, commercial, or not-for-profit sectors. 

## Data availability statement

Included data are available from the authors upon reasonable request.

## Conflicts of Interest

The authors declare that they have no conflicts of interest.
